# Leptin as well as Free Leptin Receptor Is Associated with Polycystic Ovary Syndrome in Young Women

**DOI:** 10.1155/2015/927805

**Published:** 2015-06-09

**Authors:** Nasser M. Rizk, Elham Sharif

**Affiliations:** Health Sciences Department, CAS, Qatar University, P.O. Box 2713, Doha, Qatar

## Abstract

*Background and Aim*. Leptin has two forms in the circulation: free and bound forms. The soluble leptin receptor (sOB-R) circulates in the blood and can bind to leptin. The aim of this study is to assess the concentrations of the leptin and the sOB-R in PCOS and its relation to adiposity, insulin resistance, and androgens. *Methods*. A cross-sectional study included 78 female students aged 17–25 years. Fasting serum leptin and sOB-R concentrations were measured. The anthropometric variables and the hormonal profile such as insulin, female and male sex hormones, and prolactin were assessed. *Results*. In PCOS, leptin level (ng/ml) and free leptin index (FLI) increased significantly while sOB-R (ng/ml) significantly decreased compared to control subjects. In age-matched subjects, obese PCOS had increased leptin level in ng/ml (median level with interquartile levels) of 45.67 (41.98–48.04) and decreased sOB-R in ng/ml 11.47 (7.59–16.44) compared to lean PCOS 16.97 (10.60–45.55) for leptin and 16.62 (11.61–17.96) for sOB-R with *p* values 0.013 and 0.042, respectively. However, body mass index (BMI) is significantly correlated with leptin and s-OBR, while no significant correlations with parameters of insulin resistance were detected. *Conclusion*. PCOS is associated with hyperleptinemia and increased free leptin index. Decreased sOB-R could be a compensatory mechanism for the defective action of leptin.

## 1. Introduction

The prevalence of overweight and obesity increased globally in the previous few decades [1]. Obesity and overweight have a significant influence on reproductive functions in females as the extra body fat may cause problems related to ovulatory dysfunction and infertility [2]. This abnormality is frequently associated with the polycystic ovary syndrome (PCOS). Around 4–7% of reproductive-age women have PCOS based upon the National Institute of Health (NIH) criteria [3]. About 30–75% of the patients with PCOS are obese [4] and previous data have reported that 50–60% of patients with PCOS have a central adiposity irrespective of their body mass index (BMI) [5]. There is a “complex interaction between obesity, insulin and leptin resistance, and the endocrine abnormalities in PCOS” [6].

Leptin is a product of the ob gene that acts as a sensor to the hypothalamus about the adipose tissue stores as well as a regulator of food intake and the energy balance [7]. A positive relationship between leptin and fat mass and BMI has been reported [8]. Several studies suggested that leptin may be involved in the reproductive axis function at both central and peripheral levels [9, 10]. Amenorrheic athletes have inadequate or low body fat and leptin [11]. Leptin administration in women with hypothalamic amenorrhea improves the reproduction [12]. Leptin affects the hypothalamic secretion of GnRH and gonadotropin secretion [13]. High leptin may interfere with the development of the mature oocyte and may directly activate ovarian 17-*α* hydroxylase enzyme that is involved in ovarian and adrenal steroidogenesis [13].

Leptin in the circulation is present in two main forms: a protein bound and a free form that is the biological active form [14]. The soluble leptin receptor (sOB-R) circulates in human plasma and is accomplished by binding to leptin and symbolizes the significant leptin binding activity in humans [14]. In lean subjects, leptin circulates principally in the bound form while in obesity the leptin circulates mainly as a free form due to small sOB-R concentrations [15]. The fraction of the total leptin concentrations to the sOB-R designates the free leptin index (FLI). The role of sOB-R in the reproductive system especially in PCOS is still uncertain. Few studies addressed the potential role of sOB-R in PCOS that showed inconsistent results [16–18]. In addition, conflicting results have been described on circulating leptin levels among women with different BMI, “lean, overweight, and obese women” with PCOS [19].

World Health Organization (WHO) survey in year 2010 indicated alarming levels of obesity and overweight in Qatar and it was reported that the prevalence of obesity was 38.1% while overweight was 70.2% in females [20]. In addition, no data is available about the leptin/sOB-R level among young females at the reproductive age whether they are lean or overweight/obese subjects and had clinical and biochemical features suggestive of PCOS in Qatar. The objective of this study is to explore the circulating total leptin hormone and the sOB-R levels in PCOS subjects and to assess its relationship with the metabolic and hormonal parameters in these subjects.

## 2. Subjects and Methods

### 2.1. Subjects and Study Design

A cross-sectional study was carried out from October 2011 to January 2013 at the Health Sciences Department, Qatar University. The study population consisted of 78 young women recruited randomly from Qatar University students. Questionnaire about the family history of PCOS, diabetes, infertility, and medical history such as the regularity of the menses, abnormal hair growth, or distribution was performed before the blood collection. The diagnosis of PCOS in this study is based on NIH criteria [3]. For diagnosis of PCOS, the adult female subject should present the two following criteria: menstrual irregularity such as oligomenorrhea and amenorrhea (cycle length >35 days) and hyperandrogenism (clinical by assessment of the hair growth based on the Ferriman-Gallwey score and/or biochemical such as elevated total testosterone > 2.0 nmol/L and free androgen index [FAI] > 3.8) [21]. The subjects were divided into two main groups: PCOS subjects and control non-PCOS, and each group is subdivided based upon the cutoff value of BMI of ≥25 kg/m^2^ into overweight/obese subgroup (≥25 kg/m^2^) and lean subgroup (<25 kg/m^2^). The exclusion criteria of the study included females who have thyroid disorders, Cushing syndrome, congenital adrenal hyperplasia, acromegaly, hypothalamic disorders, hyperprolactinemia and systemic inflammatory diseases and on hormonal treatment in last three months. The study received an ethical approval from Qatar University (QU-IRB 58-E/11), and an informed consent was obtained from each participant after explanation of the procedures in full detail. The informed consent and ethical guidelines were followed, based on the deceleration of Helsinki for year 2000. Diagnosis of PCOS in this study is based on NIH criteria (3).

#### 2.1.1. Study Protocols for PCOS

*(A) Anthropometric Measurements, Questionnaire, and Ferriman-Gallwey Assessment.* The weight and height were measured in kg and cm down to one decimal point, and body mass index (BMI) was calculated by dividing the weight (kg) by the square meter of height (m^2^). All participants filled out a questionnaire about their general demographic information, anthropometric measurements, and medical history (PCOS, diabetes, hypertension, and dyslipidemia and family history). In the second part, the subjects were asked about their menstrual history, diet, and lifestyle, including pharmaceutical history. The modified Ferriman-Gallwey was self-scored after explanation and demonstration in full detail to all participants. A score above 17 was considered hyperandrogenism [HA] as previously published in this geographical area [22]. Women were asked about the hair growth in a male pattern in 19 different body parts based on the modified Ferriman-Gallwey score in 2001 [23]. Ultrasound imaging was advised to those subjects who had irregular periods and/or HA to examine the ovaries for the stigma of PCOS.

*(B) Biochemical Assay.* Blood samples were collected in the follicular phase of menstrual cycles from students with regular cycles and collected independently from the day of menses from students with menstrual irregularity. Overnight fasting blood samples were drawn, followed by centrifugation, and serum was collected into aliquots and stored at −80°C for further hormonal assay. Blood glucose was measured by an enzymatic colorimetric assay in the clinical chemistry laboratory at Qatar University. The test principle of the hormonal assay was Chemiluminescent Microparticle Immunoassay (CMIA) for the qualitative determination of the following hormones: thyroid-stimulating hormone (TSH), free thyroxine (FT4) prolactin, insulin, estradiol, progesterone, testosterone, dehydroepiandrostedione sulfate (DHEA-S), and sex hormone binding globulin (SHBG). The hormonal assay was measured at the Endocrine Chemistry Lab at HMC, Qatar, by Architect. Leptin and sOB-R were measured by ELISA techniques based on the manufacturer (Cat. # RD191001100, RD194002100; Czech company BioVendor) [24]. The interassay CV% of the measured hormones was SHBG ≤ 10%, estradiol ≤ 7%, progesterone for low control ≤ 10% and for medium and high controls ≤ 7%, free T4 ≤ 8%, TSH ≤ 10%, DHEAS ≤ 10%, and testosterone ≤ 10%, prolactin ≤ 6%, and insulin ≤ 10%. The interassay CV% and intra-assay CV% were 5.5% and 8.2% for leptin and 8.7% and 7.10% for sOB-R, respectively. Free androgen index (FAI) was calculated as the total testosterone level in nmol/L over the sex hormone binding globulin (SHBG) level in nmol/L and then multiplied by 100. The free testosterone (FT) was calculated from total testosterone levels, albumin, and SHBG, using a specified formula that has been used as a diagnostic tool for hyperandrogenism [25]. The cutoff value for HOMA-IR was 2.6, and it is calculated as (fasting serum insulin (*μ*U/mL) × fasting plasma glucose (mmol L^−1^)/22.5) [26].

#### 2.1.2. Statistical Analysis

Data were explored for outliers, skewness, and normality and transformed when necessary if the normality assumption was violated. Continuous data are expressed as mean ± SD for normally distributed variables, median and interquartile range [25%–75%] for nondistributed continuous data, and number and percentage for categorical data. Two student's *t*-tests and nonparametric Mann-Whitney test were used to evaluate the differences between the continuous variables, accordingly for the analysis. Differences between categorical variables were assessed by chi-square test. ANOVA was used to evaluate the difference between three groups if the data were normally distributed and/or Wilcoxon/Kruskal-Wallis Tests (Rank Sums) test if the data were not-normally distributed were used accordingly for the analysis. Spearman correlation was used to evaluate the linear correlations between the studied variables. Partial correlation analysis with adjustment for BMI was used to assess the correlations of leptin, sOB-R with the different variables of the study. A regression analysis was performed between leptin and BMI and IR as a covariate. Odds ratios (ORs), 95% confidence interval (CI), and corresponding *p* values were analyzed by the logistic regression analysis to examine the relationship between PCOS and cofounders. The two-tailed *p* < 0.05 was considered as the cutoff value for significance. SPSS program (version 22, IBM, USA) for Mac was used for all of the statistical analysis. The graphic presentation was performed by the use of the GraphPad Prism version 6.00 for Mac (GraphPad Software, La Jolla, CA, USA, http://www.graphpad.com/).

## 3. Results

### 3.1. The Characteristics of the Study Subjects Based on the Presence of Overweight/Obesity and Nonoverweight/Nonobesity

Table 1 shows the mean and SD for age and BMI and the median values and IQ values for mFG, glucose, HOMA-IR, FAI, and hormone concentrations of estradiol, progesterone, DHEAS, SHBG, testosterone [total (T) and free (F)], insulin, and TSH of the 4 studied groups. The mean age did not differ significantly between the studied groups (*p* = 0.997). BMI was significantly higher in overweight/obese [OW/OB] subjects than in the lean subjects whether they were PCOS or non-PCOS groups, respectively (*p* < 0.0001). There was a significant difference among the studied groups (intragroup) for BMI, mFG score, testosterone (T and F), DHEAS, and FAI, as shown in Table 1.

Overweight/obese PCOS subjects had significantly higher levels of the mFG score, total testosterone, DHEAS, FAI, HOMA (IR), and free testosterone than non-PCOS overweight/obese subjects as shown in Table 1. In addition, overweight/obese PCOS subjects had significantly higher median values of the mFG score, testosterone, DHEAS, FAI, HOMA (IR), and free testosterone than other lean PCOS subjects. SHBG is significantly lower in OW/OB, OW/OB PCOS, and lean PCOS than in lean, healthy control subjects as shown in Table 1. Other variables such as prolactin, TSH, free T4, insulin, and female sex hormones are not significantly different between the various groups. The calculated HOMA-IR is significantly higher in OW/OB PCOS than in other groups without PCOS (lean and OW/OB) and lean PCOS subjects, respectively.

Furthermore, we classified the study groups in subjects with and without the insulin resistance. In addition, the frequency of IR was evident in 24/78 (30.77%) in all study subjects. IR was insignificantly higher (9/22 or 40.91%) in OW/OB subjects than in lean subjects (15/56 or 26.79%), *p* = 0.278. In addition, IR was more prevalent in OW/OB PCOS (5/8 or 62.5%) than in lean PCOS (3/10 or 30.0%) with *p* value = 0.133.

### 3.2. Serum Leptin, Soluble Leptin Receptor, and Free Leptin Index (FLI) in PCOS Based on Overweight/Obesity, the Insulin Resistance, and the Clinical Presentations of PCOS: Table 2 and Figure 1

**Figure 1:**
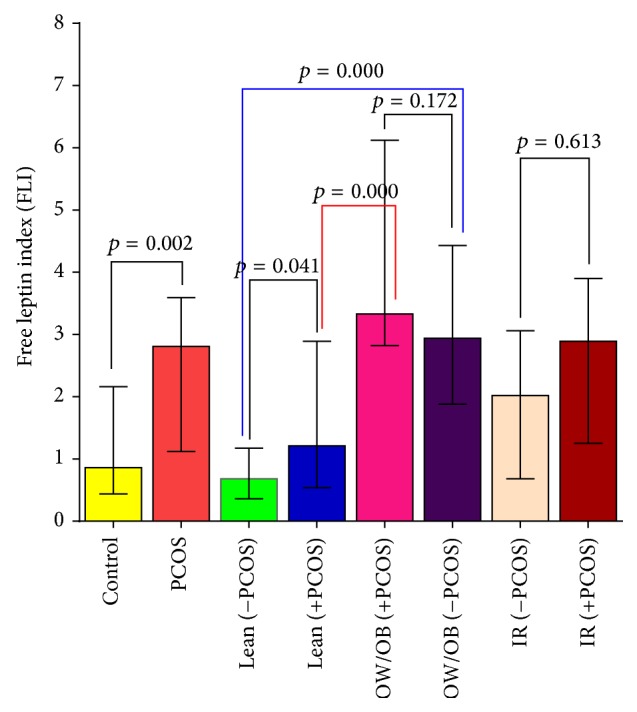
Free leptin index levels among the study subjects based on the presence of PCOS, OW/OB, and insulin resistance. Data are presented by median and interquartile (IQ) values (25%–75%). Data were analyzed by nonparametric Mann-Whitney test. *p* values are shown between different groups. Two-tailed *p*-value is significant at ≤0.05.

PCOS subjects had significantly higher median leptin levels in ng/mL (44.29) than in control non-PCOS (15.16) with *p* value = 0.013 and a significant increase of FLI (2.81) versus 0.86 with *p* = 0.001. In addition, a significant decrease of sOB-R (ng/mL) was detected in PCOS than in the control subjects with *p* value (0.002) as shown in Table 2. Furthermore, to detect the effect of overweight/obesity in PCOS subjects, OW/OB PCOS subjects had significantly higher leptin concentrations and FLI than lean PCOS subjects (*p* = 0.013; *p* = 0.000), respectively, and a significant reduction for sOB-R was detected between the two groups (*p* = 0.042), respectively. Furthermore, to evaluate effect of PCOS on leptin system in BMI-matched subjects, the results showed that PCOS in lean subjects had significantly higher FLI and reduced leptin receptor than in the results of lean subjects without PCOS (*p* = 0.002 and *p* = 0.041), respectively, while no significant difference was detected for leptin levels in both groups (*p* = 0.282). Further analysis of OW/OB subjects with and without PCOS indicated that only leptin level was significantly increased in PCOS subjects (*p* = 0.029) without significant changes in sOB-R and FLI between both groups (*p* = 0.786 and *p* = 0.172), respectively. These data are presented in Table 2 and Figure 1.

To identify the outcome of insulin resistance (IR) in PCOS, the results showed that FLI was not significantly different between both groups of PCOS with and without insulin resistance (*p* = 0.613), as illustrated in Figure 1.

### 3.3. Effect of BMI and Insulin Resistance on Leptin and Soluble Leptin Receptor in PCOS Subjects

BMI and IR are well-known factors that affect leptin level and sOB-R as shown in previous studies. We performed a multiple regression analysis to examine the effects of BMI and IR on leptin level. As shown in Table 3, only BMI is the significant predictor of leptin and sOB-R in PCOS subjects with beta unstandardized coefficients of 2.252 and −1.649 and *p* values of 0.008 and 0.000, respectively. The results demonstrated that insulin resistance (HOMA-IR) is not a significant predictor of leptin and sOB-R in PCOS subjects with beta unstandardized coefficients of −1.716 and −1.267 and *p* values of 0.287 and 0.084, respectively.

### 3.4. The Correlations between Leptin, sOB-R, and PCOS with the Endocrine Parameters and Other Variables (Table 4)

Next, we evaluated the relationship between the PCOS, plasma leptin, and sOB-R with the other parameters including the endocrine parameters and the other variables in the PCOS subjects, by partial Pearson's correlation coefficient adjusted for BMI (Table 4).

**Figure 2:**
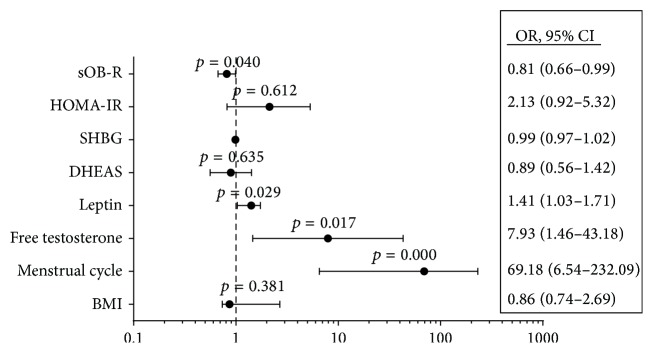
Odds ratio and (95% CI) for PCOS with the individual independent variables were analyzed by the logistic regression analysis. PCOS was diagnosed by NIH criteria. Two-tailed *p*-value is significant at ≤0.05.

Regression analysis showed a significant positive correlation between leptin and BMI (*r* = 0.588, *p* = 0.011) and a significant negative correlation between sOB-R and BMI (*r* = −0.443, *p* = 0.048) in PCOS subjects (data are not shown, in omitted figures). Therefore, BMI was included in the partial correlation analysis since it significantly associated with leptin and its soluble receptor. The partial coefficient correlation analysis revealed that leptin level had a significant positive correlation with PCOS and negative correlation with sOB-R. The soluble leptin receptor is significantly associated inversely with leptin and PCOS. PCOS is directly significantly correlated with free testosterone, DHEAS, FAI, irregular menstrual cycles, and leptin and inversely with sOB-R, as shown in Table 4. Further we calculated the odds ratio and 95% confidence intervals (CI) (Figure 2) between PCOS as a dependent variable and the following independent variables BMI and HOMA-IR in addition to the following variables: free testosterone, leptin, DHEAS, and menstrual cycle, which showed significant associations with PCOS and leptin.

The results showed that the following independent variables have a significant impact (*p* value < 0.05) on PCOS: the menstrual cycle irregularities (69.2) times, free testosterone (7.9) times, and leptin (1.33) times as shown in Figure 2.

## 4. Discussion

Leptin is an adipokine produced by the adipose tissue, and it is proposed to be one of the associates between obesity, insulin resistance, and PCOS [9, 10]. There is no single coherent model for the pathogenesis of PCOS that could explain all observed cases. The aim of this study was to evaluate the level of the circulating leptin hormone and its sOB-R in association with the clinical and metabolic parameters of PCOS subjects among the female students in Qatar University. This correlation can explore the role of leptin and its soluble receptor in women with PCOS. The current findings of this study showed that leptin hormone and the free leptin index are significantly higher, and sOB-R is significantly lower in all PCOS subjects compared to the healthy control of age-matched subjects. Moreover, age-matched overweight/obese PCOS females showed that leptin and free leptin index are significantly higher than in lean PCOS subjects while they showed opposite results for sOB-R. In addition, PCOS significantly increases the free leptin index and decreases the leptin receptor in lean subjects. Moreover, in age- and BMI-matched OW/OB subjects, only the leptin increases in OW/OB PCOS subjects without significant effects on sOB-R and FLI. The present data also demonstrated that leptin and soluble leptin receptor are significantly associated with PCOS independently of IR and it is dependent on BMI. Overweight/obese females had higher levels of leptin and biochemical parameters of hyperandrogenism including testosterone and DHEAS and reduced sOB-R. Leptin, sOB-R, testosterone, and menstrual irregularities are the significant independent factors for PCOS among the study subjects. The implications of these findings will be discussed in the next paragraphs.

Earlier studies on leptin levels in PCOS subjects are still controversy [27–30]. Several studies reported increased circulating leptin concentrations in PCOS and suggested that leptin has a role in its pathogenesis [31, 32], which are similar to the findings of the current study. In addition, Vicennati et al. (1998) reported an increased leptin level in obese PCOS subjects compared to lean PCOS subjects [32]. Another study by Panidis et al. (2003) demonstrated an increase in leptin level in overweight women with PCOS and insulin resistance [28]. A recent study by Olszanecka-Glinianowicz et al. (2013) demonstrated higher leptin levels in PCOS subjects compared with age- and BMI-matched control group. Furthermore, they observed more elevated leptin in obese subjects than in lean PCOS subjects [33]. Other studies showed elevated leptin level in obese PCOS patients [34, 35], which is consistent with the data of the current study as shown in Figure 2. On the contrary, several studies showed no significant difference of leptin between subjects with and without PCOS [36, 37]. The difference in such studies with the current data could be due to the sample size, type of sample collection, and the heterogeneity nature of the PCOS syndrome. Previous studies evaluated the role of the soluble leptin receptor in PCOS subjects [16, 30]. Hahn et al. (2006) demonstrated a significant stepwise increase in leptin, with a significant stepwise reduction in sOB-R from lean to overweight to obese subjects, respectively. The investigator also reported that lean PCOS patients had lower sOB-R levels and higher free leptin indices, compared with BMI-matched lean controls, respectively [30]. These data are consistent with the findings of the current study where (1) sOB-R levels were reduced in all PCOS subjects compared to BMI-matched healthy controls. In addition, in overweight and obesity PCOS subjects, the sOB-R level decreases and FLI increases significantly compared with lean PCOS subjects. Moreover, in lean PCOS subjects, the sOB-R level decreases significantly compared to lean control (−PCOS) subjects while FLI showed opposite effects. Similar findings were demonstrated in PCOS patients from different countries [27, 36]. Contrary to the current data, Sepilian et al. (2006) demonstrated no significant change in leptin, sOB-R, and FLI between PCOS women with insulin resistance and BMI-matched controls [16], which is not consistent with the current findings. The difference in such study with the present data could be due to the sample size, type of sample collection, and the heterogeneity nature of the PCOS syndrome.

We also assessed the possible association between the leptin system (hormone and its soluble receptor) and the hormonal profile, BMI, and the menstrual irregularities. Only a significant association was detected between BMI, leptin, and leptin receptor as shown in previous studies [30, 38]. Moreover, the current data demonstrated that soluble leptin receptor is negatively correlated with leptin, as reported in previous studies [30, 39]. The correlation between total leptin and leptin receptor in PCOS cohort with the female gonadal, thyroid, and androgen hormones failed to detect any significant correlations after adjusting BMI. Such findings are in agreement with previous studies [30, 36] though previous studies reported a significant correlation between DHEAS levels and the free leptin index [16]. Conflicting results regarding the correlations between androgen and estradiol with the leptin were observed in other studies [16, 40]. The controversy could be due to differences in the subject populations and the sample size and the heterogenic nature of the PCOS syndrome.

Hyperinsulinemia and insulin resistance play a critical role in the development of the polycystic ovary syndrome (PCOS). Insulin upregulates leptin mRNA in adipocytes, signifying its potential role in leptin secretion [41]. On the contrary, leptin inhibits insulin-mediated activation of gonadotropin on steroidogenesis [42]. Insulin resistance and hyperinsulinemia are present in up to 65% of obese women with PCOS and in up to 20% of lean women with PCOS [43]. The high leptin in hyperinsulinemia of PCOS women may be a secondary consequence of insulin-stimulated leptin synthesis. Several studies showed that leptin is correlated with insulin level and insulin resistance in PCOS subjects [34, 44], though other studies demonstrated no correlation [16, 32]. The data of the current study did not illustrate any noteworthy correlation between insulin and leptin and HOMA with leptin and sOB-R. The controversy could be attributed to the differences in the sample size, the study populations, and the heterogenic nature of the PCOS syndrome.

In obesity, leptin resistance or inadequate leptin action is indicated by high leptin level [45], and several mechanisms were postulated to explain the defect of leptin action in obese subjects [46]. Hahn et al. (2006) hypothesized that the reduction of sOB-R is a tentative mechanism to overcome the leptin resistance or the defective leptin action [30]. In the current study, PCOS and the obesity status increases leptin level and decresaes the soluble leptin receptor. To gain insight into these findings, we evaluated the impact of both factors, the body mass index as a representative of the obesity and the insulin resistance calculated by HOMA-IR in the current study by regression analysis on leptin and sOB-R levels. The current data indicated that hyperleptinemia and decreased sOB-R are independent of insulin resistance and only related to the BMI and adiposity of the PCOs subjects. These findings are supported by earlier studies [17, 30], which showed correlations of leptin with adiposity independent of insulin resistance. Thus, the correlation between the BMI and leptin and its sOB-R observed in this study and in the previous studies may indicate that adiposity (BMI) may represent the primary determinant of the leptin system in PCOS, as well as being known in the general populations. Thus, it could be concluded that the higher incidence of obesity, rather than hyperinsulinemia, could be a confounding factor in hyperleptinemia observed in OW/OB PCOS patients. Furthermore, the current data showed that even with BMI-matched subjects, in lean and in OW/OB subjects with and without PCOS leptin levels, sOB-R and FLI were significantly different. Such findings could also highlight a direct role of leptin in the pathogenesis of PCOS independent of obesity/BMI and insulin resistance and other hormonal factors such as androgens in the current study.

The reduction of sOB-R in PCOS could be a tentative mechanism to overcome the defect of leptin action in these patients [30]. The difference in androgen levels in the studied subgroups could explain the current results for leptin and sOB-R and FLI in BMI-matched subjects as shown in previous studies [28, 47]. The genetic background variability and the heterogenicity of PCOS could be another factor for the variation of leptin and its soluble receptor in the BMI-matched PCOS subjects [48]. Our data may require further studies to evaluate the role of leptin and the soluble receptors in a well-characterized large cohort. It seems that heterogenicity of PCOS could explain different mechanisms for leptin's action on the reproductive system in these patients.

The current data showed that the leptin and sOB-R with testosterone and menstrual irregularities are the significant factors contributing to the PCOS in the present study. The current finding highlights that leptin and sOB-R could be independent factors in the pathogenesis of the PCOS, independent of BMI. Several studies suggested a different mechanism through which leptin could affect the reproductive functions [9, 10]. Low leptin level in amenorrheic athletes with low body fat [11] and the leptin application in females with hypothalamic amenorrhea restore the reproduction [12]. Leptin may affect the central secretion of GnRH and gonadotropin from the hypothalamus [13]. In addition, hyperleptinemia may inhibit the development of the mature oocyte development directly and affect ovarian and adrenal steroidogenesis [13]. Defective leptin action that is observed in PCOS subjects and its consequences may be explained by the impaired central action of leptin in the hypothalamus with more weight gain and adiposity and/or peripherally via the impaired action of leptin on mature ovum production. The soluble leptin receptor (sOB-R) that circulates in human plasma is capable of binding to leptin that could protect against obesity [14]. The role of sOB-R in the reproductive system especially in PCOS could be explained to compensate for the defective leptin action observed in PCOS subjects whether lean or obese as suggested by another study [30].

The present study has some limitations, such as the small size of the studied samples, the narrow range for the age of the studied subjects, and the cross-sectional nature of the study. Further studies are needed with large sample size, including a wide variety of ages and occupations and of longitudinal nature. This could help to understand the detailed associations and actions of insulin and androgen on leptin, sOB-R, and FLI in PCOS.

In conclusion, PCOS is associated with hyperleptinemia, decreased sOB-R, and increased free leptin index. Decreased sOB-R could be a compensatory mechanism for the defective action of leptin. Elevated serum leptin in PCOS women could be due to the positive correlation between leptin and BMI and is independent of insulin resistance. Leptin and its soluble receptor are implicated in the pathophysiology of PCOS that needs further investigations.

## Figures and Tables

**Table 1:** Clinical and biochemical characters of the study subject groups: overweight/obese (OW/OB), lean, lean with PCOS, and overweight/obese (OW/OB) PCOS subjects.

Variables	Non-PCOS (*n* = 60)		PCOS (*n* = 18)		*p* value
	Lean (*n* = 14)	OW/OB (*n* = 46)	Lean + PCOS (*n* = 10)	OW/OB + PCOS (*n* = 8)	
Age (years)	21.00 (1.30)	21.50 (1.59)	21.10 (1.85)	21.25 (1.49)	0.997
BMI (kg/m^2^)	20.867 (2.41)	28.93 (3.74)^*α*^	20.93 (3.03)	28.64 (2.89)^*∗*^	<0.0001
mFG score	14.00 (11.00–15.00)	11.50 (8.00–15.50)	13.0 (9.5–19.8)	15 (13.8–16.5)^*∗*∞^	<0.0001
Glucose (mg/dL)	89.00 (82.00–95.00)	92.00 (79.00–97.25)	85.5 (79.81–99.32)	95 (89.31–101.56)	0.689
Estradiol (pmol/L)	323.00 (145.00–522.00)	156.00 (124.00–223.50)	194 (139.80–294.80)	221 (80.00–442.00)	0.676
Progesterone (nmol/L)	0.60 (0.50–2.00)	1.40 (0.40–4.25)	0.6 (0.45–10.50)	0.9 (0.73–8.00)	0.946
Testosterone (nmol/L)	1.02 (0.52–1.66)^*∗*^	1.26 (0.53–1.98)^*α*^	1.67 (0.91–2.77)^≠^	2.77 (2.10–4.05)^*∗*∞^	<0.0001
DHEAS (*μ*mol/L)	6.69 (5.35–8.77)^*∗*^	7.87 (6.55–8.55)^*∗*^	8.06 (5.18–10.16)	11.51 (10.23–14.71)^*∗*∞^	<0.0001
SHBG (nmol/L)	57.00 (45.50–72.50)	37.00 (33.00–65.00)^*α*^	33 (28.5–50.5)^≠^	36 (18.75–60.75)	0.279
FAI	1.49 (0.72–3.33)	1.74 (1.13–3.71)^*α*^	5.3 (2.4–10.9)^*∞*≠^	10.8 (3.8–14.6)^*∗*∞^	<0.0001
Free testosterone (nmol/L)	0.010 (0.005–0.021)^*∗*^	0.018 (0.008–0.028)^*α*^	0.032 (0.015–0.057)^*∞*≠^	0.059 (0.027–0.079)^*∗*∞^	<0.0001
Prolactin (mIU/L)	306.00 (223.0–377.0)	300.50 (244.0–372.7)	368 (289–397.5)	375 (213.1–547.0)	0.419
Insulin (*μ*U/mL)>	11 (6.5–23.00)	11 (6.5–23.00)	10 (5.5–32.5)	28.5 (13.3–43.0)	0.327
TSH (mIU/L)	1.20 (0.95–2.40)	1.20 (0.95–2.40)	1.36 (1.07–2.44)	1.73 (1.08–3.39)	0.069
Free T4 (pmol/L)	14.20 (12.80–14.60)	14.20 (12.80–14.60)	15.1 (13.4–16.5)	14.2 (10.5–14.5)	0.560
HOMA (IR)	2.05 (1.26–5.30)	2.05 (1.26–5.30)	3.8 (2.91–7.62)^≠^	8.6 (2.24–13.95)^*∗*∞^	0.799

Data are presented by median and interquartile (IQ) values (25%–76%). Data were analyzed by nonparametric Wilcoxon/Kruskal-Wallis test for all group and pair-test with nonparametric comparisons for each pair using Wilcoxon method. OW/OB (overweight/obese); body mass index (BMI); mean modified Ferriman-Galleway (mFG); free androgen index (FAI); thyroxin (T4); dehydroepiandrosterone sulfate (DHEAS); thyroid-stimulating hormone (TSH); sex hormone binding globulin (SHBG). *p* value for comparison of all groups. OW/OB PCOS is significantly different from lean PCOS with ^*∗*^ *p*, OW/OB PCOS is significantly different from OW/OB Non-PCOS with ^*∞*^ *p*, OW/OB Non-PCOS is significantly different from lean Non-PCOS with ^*α*^ *p*, and Lean Non-PCOS is significantly different from lean PCOS with ^≠^ *p*. Two-tailed *p* value less than 0.05 (<0.05) is significant.

**Table 2:** Leptin levels (ng/mL), sOB-R (ng/mL), and LFI among PCOS subjects and in the presence/absence of overweight/obesity.

	PCOS [*n* = 18]	Control [*n* = 60]	*p*
Leptin (ng/mL)	44.29 (16.14–47.78)	15.16 (11.87–36.27)	0.013
sOB-R (ng/mL)	14.80 (11.11–17.20)	20.06 (14.75–31.85)	0.002
FLI	2.81 (1.12–3.59)	0.86 (0.44–2.16)	0.001
BMI kg/m^2^	24.36 (4.88)	22.88 (4.48)	0.234

	Lean (+PCOS) [*n* = 10]	OW/OB (+PCOS) [*n* = 8]	*p*

Leptin (ng/mL)	16.97 (10.60–45.55)	45.67 (41.98–48.04)	0.013
sOB-R (ng/mL)	16.62 (11.61–17.96)	11.47 (7.59–16.44)	0.042
FLI	1.21 (0.54–2.89)	3.33 (2.82–6.12)	0.000

	Lean (+PCOS) (*n* = 10)	Lean (−PCOS) [*n* = 46]	*p*

Leptin (ng/mL)	16.97 (10.60–45.55)	14.32 (10.52–24.93)	0.282
sOB-R (ng/mL)	16.62 (11.61–17.96)	23.69 (17.49–36.76)	0.002
FLI	1.21 (0.54–2.89)	0.68 (0.37–1.171)	0.041

	OW/OB (+PCOS) [*n* = 8]	OW/OB (−PCOS) [*n* = 14]	*p*

Leptin (ng/mL)	45.67 (41.98–48.17)	39.18 (20.53–44.77)	0.029
sOB-R (ng/mL)	11.47 (7.59–16.44)	12.02 (9.09–15.26)	0.768
FLI	3.33 (2.82–6.12)	2.94 (1.88–4.43)	0.172

Data are presented by median and interquartile (IQ) values (25%–76%) and BMI as mean and SD. Data were analyzed by nonparametric Wilcoxon/Kruskal-Wallis test. Two-tailed *p* value less than 0.05 (<0.05) is significant.

**Table 3:** The correlation between serum leptin, sOB-R, and both BMI and HOMA-IR in PCOS subjects.

Model	Unstandardized coefficients		Standardized coefficients		
	*B*	Std. error	Beta	*t*	*p*
Leptin					
(Constant)	−17.846	17.940		−0.995	0.336
HOMA	−1.716	1.553	−0.230	−1.105	0.287
BMI	2.252	0.742	0.631	3.034	0.008
sOB-R					
(Constant)	63.849	7.452		8.567	0.000
HOMA	−1.267	0.699	−0.175	−1.812	0.074
BMI	−1.649	0.316	−0.505	−5.222	0.000

Dependent variables (constant): leptin (ng/mL) and sOB-R (ng/mL) in order. Regression analysis was used to analyze the effect of HOMA-IR and BMI on leptin and sOB-R in PCOS subjects. Two-tailed *p* value less than 0.05 (<0.05) is significant.

**Table 4:** The partial coefficient correlation of PCOS, leptin, and sOB-R in PCOS subjects.

Variable	PCOS		Leptin ng/mL		Soluble leptin receptor	
	Coefficient *r*	*p*	Coefficient *r*	*p*	Coefficient *r*	*p*
Testosterone (F)	0.745	<0.0001	0.416	0.085	−0.148	0.557
DHEAS	0.411	0.0002	0.406	0.094	−0.082	0.744
Progesterone	0.020	0.865	0.154	0.494	0.078	0.494
Estradiol	0.140	0.204	0.124	0.554	−0.010	0.926
Insulin	0.155	0.176	0.282	0.203	−0.228	0.064
SHBG	−0.171	0.445	−0.367	0.235	0.198	0.081
Glucose	0.011	0.892	0.199	0.086	0.044	0.703
FAI	0.685	0.003	0.353	0.150	0.193	0.055
Prolactin	0.081	0.480	0.180	0.115	0.034	0.765
TSH	0.016	0.893	0.131	0.561	−0.234	0.039
Free T4	0.092	0.577	−0.051	0.757	−0.028	0.872
Irregular cycles (yes)	0.580	0.000	0.143	0.212	−0.255	0.086
PCOS (yes)	—	—	0.282	0.012	−0.349	0.001
Leptin	0.277	0.014	—	—	−0.361	0.001
sOB-R	−0.313	0.005	−0.718	0.001	—	—
HOMA-IR	0.254	0.247	0.323	0.124	−0.353	0.074

Data were analyzed by partial coefficient correlation after adjustment for BMI. Correlation is significant at two-tailed *p* value at <0.05. Body mass index (BMI); free androgen index (FAI); thyroxin (T4); dehydroepiandrosterone sulfate (DHEAS); thyroid-stimulating hormone (TSH); sex hormone binding globulin (SHBG); soluble leptin receptor (sOB-R); free leptin index (FLI); free testosterone (F); and insulin resistance (HOMA-IR).
